# Vitamin D Deficiency in Adults With Tibial Plateau Fractures: A Comparative Analysis Using a Large National Population

**DOI:** 10.7759/cureus.99974

**Published:** 2025-12-23

**Authors:** Constantinos D Apostolou, Georgios Chatzipanagiotou, Nikolaos Papazotos, Efstathios Chronopoulos

**Affiliations:** 1 Orthopedics and Trauma, Evangelismos General Hospital, Athens, GRC; 2 Orthopedic Surgery, National and Kapodistrian University of Athens, Athens, GRC; 3 Orthopedic Surgery, Konstantopoulio General Hospital, Athens, GRC

**Keywords:** bone metabolism, orthopedic trauma, tibial plateau fracture, vitamin-d deficiency, vitamin-d supplements

## Abstract

Background and aim

Vitamin D is essential for bone metabolism and fracture prevention, yet its status in tibial plateau fractures remains underexplored. This study aimed to evaluate serum vitamin D levels in patients with tibial plateau fractures and compare them with data from the general Greek population, investigating whether vitamin D deficiency is more prevalent among fracture patients.

Methods

We prospectively evaluated 45 adults presenting with tibial plateau fractures at a tertiary hospital in Greece. Serum 25-hydroxyvitamin D (25{OH}D) was measured on admission and categorized as sufficient (>30 ng/mL), insufficient (20-30 ng/mL), or deficient (<20 ng/mL). Values were compared with published data from 8780 individuals from the Greek general population. Statistical analysis was performed between these two groups.

Results

Patients had markedly lower mean vitamin D levels than the population sample (16.24±8.21 ng/mL vs. 25.08±14.40 ng/mL; p<0.0005). Deficiency was strikingly more common in the fracture group (75.6% vs. 40%; p<0.0005). Both groups were similar with respect to age but not in gender, since in the general population, the majority of the subjects were female compared with the male dominance in the tibial plateau fracture group (73.3% vs. 30%; p<0.0005).

Conclusion

Adults with tibial plateau fractures show profound vitamin D deficiency compared with the general Greek population. Screening and supplement treatment may be advisable in this patient group. Further studies should investigate whether low vitamin D could be a risk factor for tibial plateau fracture.

## Introduction

Vitamin D deficiency has been increasingly recognized as a critical factor affecting bone health and fracture healing [1]. Beyond its established role in calcium-phosphate homeostasis and bone metabolism, vitamin D exerts pleiotropic effects on numerous cellular processes involved in inflammation, osteogenesis, and myogenesis [2]. Through activation of the vitamin D receptor (VDR), vitamin D regulates more than 3000 genes implicated in bone remodeling, immune modulation, and tissue repair [2]. Deficiency disrupts these mechanisms, leading to impaired osteoblastic differentiation, delayed callus mineralization, and heightened inflammatory responses, collectively hindering bone repair and wound regeneration [2,3].

Despite its biological importance, vitamin D deficiency remains a global health issue [4]. According to the Endocrine Society and the Institute of Medicine, vitamin D sufficiency is defined as ≥30 ng/mL, insufficiency as 20-30 ng/mL, and deficiency as <20 ng/mL [5]. Approximately 40% of Europeans are deficient. Notably, even in sun-rich regions, such as Southern Europe, deficiency rates remain high due to limited outdoor exposure, dietary inadequacy, and lifestyle factors [6].

Vitamin D deficiency is of particular concern among older adults, as reduced bone mineral density and muscle weakness often manifest clinically as hip and other non-vertebral fractures [7]. Several studies have demonstrated that combined calcium and vitamin D supplementation can effectively reduce the risk of such fractures [7,8]. While earlier research primarily concentrated on elderly cohorts, more recent investigations indicate that vitamin D insufficiency and deficiency remain highly prevalent across broader age groups, including adults and adolescents.

Orthopedic trauma patients are particularly vulnerable due to immobilization, limited sun exposure, and pre-existing deficiency [8]. Several studies have demonstrated that hypovitaminosis D is common among patients with fractures or complex musculoskeletal injuries and may contribute to delayed healing, nonunion, and poorer postoperative outcomes [9,10].

This study aimed to assess the prevalence of vitamin D deficiency among adults presenting with tibial plateau fractures and to compare these findings with data from the general Greek population, thereby contextualizing them within the broader European literature.

## Materials and methods

This prospective observational study was conducted at the orthopedic clinic of a tertiary hospital in Greece over an 11-month period, from March 2024 to February 2025. Forty-five consecutive adults (≥18 years) with acute tibial plateau fractures, who presented to the hospital’s emergency department and were confirmed by radiography and/or CT, were included. Patients with pathological fractures, metabolic bone diseases, and active vitamin D supplementation were excluded. The study protocol received approval from the hospital's ethics committee, and informed consent was obtained from all participants prior to their inclusion. Demographic data (age, sex), fracture classification, and serum 25(OH)D levels were recorded on the day of hospital admission. Serum 25(OH)D was measured using chemiluminescent immunoassay and categorized as sufficient (>30 ng/mL), insufficient (20-30 ng/mL), or deficient (<20 ng/mL) [5].

Population reference data were drawn from a large epidemiologic study of 8780 Greek adults reporting a mean 25(OH)D of 25 ng/mL and a deficiency prevalence of 39.9% [6]. The data for this study were collected from the Nuclear Medicine Department at the Tertiary Healthcare Center, AHEPA University Hospital, in Thessaloniki, Greece. All individuals with vitamin D measurements at the respective laboratories from January 2013 to October 2017 were included in this study. Data regarding 25(OH)D levels and other patient data, including sex, age, and the month at which the blood sample was drawn, were collected. Individuals with more than three missing data values were excluded. In the reference population, for 112 subjects, sex was not recorded.

Statistical analysis

Quantitative variables were expressed as mean±standard deviation (SD), whereas qualitative variables were presented as frequencies (n) and percentages (%). Comparisons between the study group (patients with tibial plateau fractures) and the reference population were performed using the independent samples t-test for continuous variables and the chi-square test or Fisher’s exact test, as appropriate, for categorical variables. All statistical analyses were performed using IBM SPSS Statistics version 21.0 (Armonk, NY: IBM Corp.) for Windows. All tests were two-tailed, and p<0.05 was considered statistically significant.

## Results

Forty-five patients (mean age: 48.9±13.2 years; 73.3% male) with tibial plateau fractures were analyzed. Compared with 8780 individuals from the general population, the fracture cohort had significantly lower vitamin D concentrations (16.2±8.2 vs. 25.0±14.4 ng/mL; p<0.0005) (Table 1).

**Table 1:** Comparison between fracture patients and Greek population.

Variables	Fracture group (n=45)	Greek population (n=8780)	p-Value
Vitamin D status - sufficient	3 (6.7%)	2363 (26.9%)	0.001
Vitamin D status - insufficient	8 (17.8%)	2913 (33.2%)	0.02
Vitamin D status - deficient	34 (75.6%)	3504 (39.9%)	<0.0005
25(OH)D (ng/mL), mean±SD	16.2±8.2	25.0±14.4	<0.0005
Age (years), mean±SD	48.9±13.2	49.3±20.0	0.904

**Figure 1:**
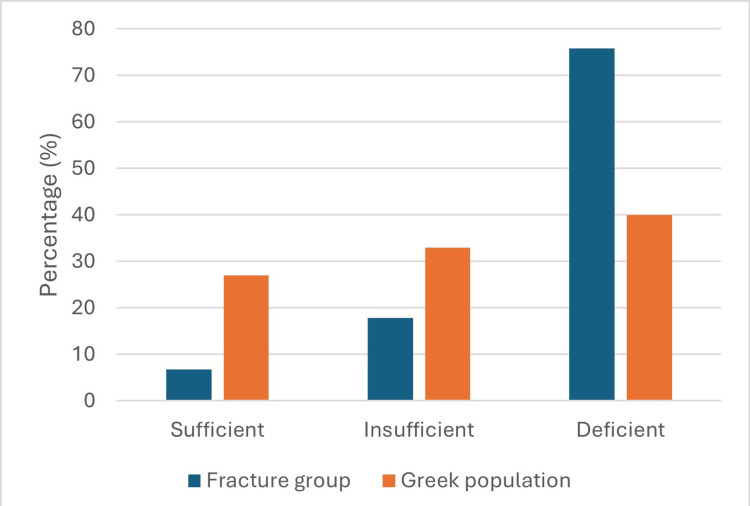
Comparison of vitamin D status between fracture patients and the general population.

**Figure 2:**
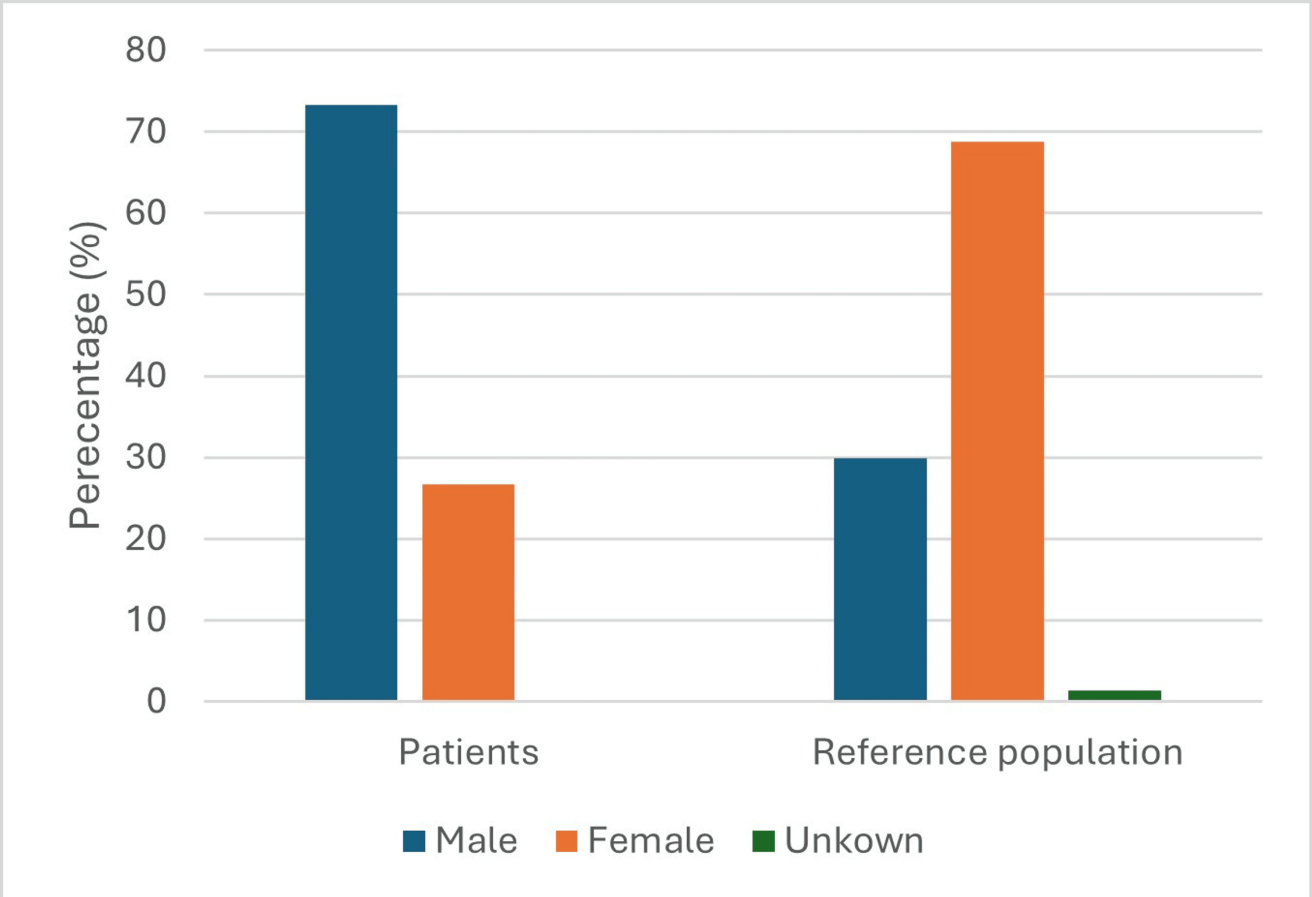
Comparison of gender in the two groups.

Vitamin D deficiency was significantly more common in the fracture group (75.6% vs. 39.9%; p<0.0005), while sufficiency was rare (6.7% vs. 26.9%) (Figure 1). There was no difference in age (p=0.904), but men predominated in the fracture group (p<0.0005) (Figure 2).

## Discussion

The present study demonstrates that adults presenting with tibial plateau fractures have a significantly higher prevalence of vitamin D deficiency compared with the general Greek population [6]. The association between vitamin D deficiency and bone fragility has been extensively documented. Low 25(OH)D levels impair calcium absorption, disrupt osteoblastic activity, and weaken bone microarchitecture, leading to increased susceptibility to fractures and delayed consolidation [11-13]. Similarly, in one of our studies, we demonstrated that patients with tibial plateau fractures frequently exhibit underlying metabolic bone disturbances, supporting the notion that low 25(OH)D concentrations contribute to impaired bone quality and structural fragility [14]. Other than this report, the literature regarding the relation between tibial plateau fractures and vitamin D deficiency is extremely limited.

In comparison with our findings, similar studies from other geographic regions report comparably high prevalences of hypovitaminosis D among orthopedic trauma patients. Johnson et al. investigated patients with acute hip fractures in a “sunny climate” (Southern California) and found mean serum 25(OH)D levels of approximately 26.4 ng/mL in fracture patients vs. 29.9 ng/mL in elective joint surgery controls [15]. Notably, more than 65% of fracture patients were vitamin D insufficient or deficient, despite living in a region with high annual sunlight exposure. These data reinforce that sunlight availability alone does not guarantee adequate vitamin D status, likely due to behavioral, cultural, or physiological factors that limit dermal synthesis.

Likewise, Bee et al. evaluated orthopedic trauma patients in the Northwestern United States and reported persistently low vitamin D levels across all seasons [16]. Even during periods of increased sunlight, the majority of patients remained below sufficiency thresholds. These observations underscore that geographic latitude, seasonal variation, and lifestyle collectively modulate vitamin D status; however, none appear sufficient to prevent widespread insufficiency in trauma populations.

Similar results have been reported by Ko et al., who found that vitamin D supplementation improved functional outcomes in patients with osteoporotic vertebral compression fractures [17]. These findings reinforce the crucial role of vitamin D in maintaining skeletal integrity, promoting bone mineralization, and supporting the physiological process of fracture healing [1,3,4].

While our study design and results initially appear comparable to those reported in previous studies, the key distinction lies in the focus on a patient group with a specific fracture type that is not typically classified as a major osteoporotic fracture. Consequently, the observed differences were less anticipated. Additionally, due to the nature of the fracture under investigation, the patient cohort had a relatively lower mean age, which facilitated a more precise comparison with the general population.

Previous studies have also demonstrated the beneficial effects of vitamin D on articular cartilage and in cases of severe soft-tissue injury [2,18]. Therefore, it is reasonable to assume that patients with the specific type of fracture examined in our study could benefit in multiple postoperative aspects if vitamin D levels are corrected, given that this fracture predisposes to post-traumatic knee osteoarthritis and is often associated with significant soft-tissue damage.

Beyond its skeletal actions, vitamin D also influences muscle function, postural balance, and fall risk factors that indirectly affect fracture incidence and recovery [1]. Deficiency may therefore not only compromise bone architecture but also delay functional rehabilitation following lower limb trauma. This dual impact on bone and muscle physiology is particularly relevant in elderly patients and those with limited mobility after orthopedic injury [19].

Integrating vitamin D assessment into the standard diagnostic workup of fracture patients could provide clinicians with valuable information on bone metabolic health and guide personalized interventions to optimize recovery and minimize the risk of refracture. Finally, further studies should investigate whether low vitamin D could be a risk factor for tibial plateau fracture.

Limitations

This study has certain limitations that should be acknowledged. The single-center design and relatively small sample size may limit the generalizability of our findings. Additionally, seasonal variations, sunlight exposure, and individual supplementation habits were not systematically assessed and could have influenced vitamin D status. Finally, the comparison with national reference data was based on a previously published report, which may differ in methodology and population characteristics. Nevertheless, it is reassuring that no statistically significant difference was found between the two groups regarding age, as older age is a known risk factor for vitamin D deficiency. In contrast, although a difference was observed in sex distribution, sex has not been shown to substantially influence vitamin D levels [20]. Despite these limitations, the present study provides valuable insights into the metabolic bone status of patients with tibial plateau fractures in a Mediterranean cohort. Additionally, this study is expanding the very limited published literature regarding the relation between this specific fracture and vitamin D deficiency.

## Conclusions

Adults with tibial plateau fractures in this study were found to exhibit markedly lower vitamin D levels and a higher prevalence of deficiency compared with the general population. Routine screening and correction of vitamin D deficiency may enhance bone healing and recovery in this challenging orthopedic injury. Given the complexity of this injury, typically associated with considerable soft tissue damage and articular cartilage defects, this intervention may be of considerable clinical importance.
